# Food Communication and its Related Sentiment in Local and Organic Food Videos on YouTube

**DOI:** 10.2196/16761

**Published:** 2020-08-10

**Authors:** Xanat Vargas Meza, Toshimasa Yamanaka

**Affiliations:** 1 Department of International Industrial Engineering National Institute of Technology Ibaraki College Hitachinaka Japan; 2 Graduate School of Comprehensive Human Sciences University of Tsukuba Tsukuba Japan

**Keywords:** social networks, framing, semantic analysis, sentiment analysis, organic, local, food, YouTube

## Abstract

**Background:**

Local and organic foods have shown increased importance and market size in recent years. However, attitudes, sentiment, and habits related to such foods in the context of video social networks have not been thoroughly researched. Given that such media have become some of the most important venues of internet traffic, it is relevant to investigate how sustainable food is communicated through such video social networks.

**Objective:**

This study aimed to explore the diffusion paths of local and organic foods on YouTube, providing a review of trends, coincidences, and differences among video discourses.

**Methods:**

A combined methodology involving webometric, framing, semantic, and sentiment analyses was employed.

**Results:**

We reported the results for the following two groups: organic and local organic videos. Although the content of 923 videos mostly included the “Good Mother” (organic and local organic: 282/808, 34.9% and 311/866, 35.9%, respectively), “Natural Goodness” (220/808, 27.2% and 253/866, 29.2%), and “Undermining of Foundations” (153/808, 18.9% and 180/866, 20.7%) frames, organic videos were more framed in terms of “Frankenstein” food (organic and local organic: 68/808, 8.4% and 27/866, 3.1%, respectively), with genetically modified organisms being a frequent topic among the comments. Organic videos (N=448) were better connected in terms of network metrics than local organic videos (N=475), which were slightly more framed regarding “Responsibility” (organic and local organic: 42/808, 5.1% and 57/866, 6.5%, respectively) and expressed more positive sentiment (M ranks for organic and local organic were 521.2 and 564.54, respectively, Z=2.15, *P*=.03).

**Conclusions:**

The results suggest that viewers considered sustainable food as part of a complex system and in a positive light and that food framed as artificial and dangerous sometimes functions as a counterpoint to promote organic food.

## Introduction

### Background

Sustainability has been receiving global attention with the United Nations promotion of Sustainable Development Goals. Goal 2 “Zero Hunger” involves food security and agriculture development [1]. In these regards, organic and local foods have been proposed as alternatives to industrialized patterns of food production, consumption, and distribution, with the potential to overcome the structural issues of mainstream sustainable consumption practices [2]. However, sustainable food markets still need to address a lack of information on resource efficiency and eating habits [3].

The global organic food market size was estimated at US $90 billion in 2016, with the United States, Germany, and France being the main consumers and India, Uganda, and Mexico being the main producers [4]. Lee and Yun [5] provided a model to explain organic food consumer behavior based on stimuli (nutrition, nature, ecological welfare, sensory appeal, and price), organism (utilitarian and hedonic attitudes), and response (buying intention). In the European context, animal welfare, regional production, and fair price were important in purchase decisions [6].

On the other hand, the marketing of food products showed a change in the 2000s from industrialized processes to artisanal, small, and locally based processes [7]. In 2007, the term “locavores” was used for those who eat food produced within a 100-mile radius [8]. Darby et al [9] found that a consumer’s will to pay for local foods is independent from values associated with product freshness and farm size. Additionally, local food is linked to authenticity in tourism contexts [10]. These findings suggest that “organic” and “local” are conceptually different enough to be distinguishable for consumers, although the terms frequently overlap in food descriptions. However, it is important to understand consumers’ definitions, attitudes, and behaviors related to sustainable food in the context of modern technologies, such as social networking sites.

There is evidence that sensationalist and erroneous content is being fueled by social media search engines owing to their business focus [11,12]. How this impacts the communication of sustainable food products is largely unknown. Thus, this study aimed to explore organic and local food products on YouTube, with the following objectives:

- To find diffusion paths of local and organic food products on YouTube by collecting information on related videos and comparing their network levels with social network analysis.
- To review trends and differences among discourses through framing analysis on video content.
- To explore the opinions, attitudes, behaviors, and emotions expressed by viewers through semantic and sentiment analyses on comments extracted from the videos.

### Literature Review

With the advent of internet 2.0, online collaboration and activism increased, transforming the internet into a conversational space through the rise of social networking sites. Internet 3.0 incorporated location and real-time aware devices and apps, prompting more personalization of products and services.

Social networks can reduce the communication gap between producers, consumers, and other interested people. For example, business engagement on Twitter is related to consumers’ web-based word-of-mouth communication, and its influence reaches consumers with a second-degree relationship to brands [13]. However, Rutsaert et al [14] argued that social media can contain inaccurate, incorrect, or misleading information and that there is a delicate balance between fast communication in a food crisis and amplification of risk perceptions.

Previous social media studies on food communication through text included two-way communication by public organizations related to food safety and nutrition, and it was found that the main themes were queries and complaints, benefits of social media in query and complaint services, content redesign driven by social media use, and social media to learn about consumers [15]. Pantelidis [16] examined restaurant reviews, arguing that costumers considered food, service, ambience, price, menu, and décor regardless of their economic conditions. Moreover, direct-to-consumer marketing strategies of farmers on Facebook were positively related to their social capital and farm revenue [17].

Structural and social factors of web-based communication channels affect their roles in the image construction process of organic food brands [18]. Studies on organic food communication and eating habits expressed by Koreans and Mexicans on Twitter revealed that (1) Mexicans focused on basic food products in street markets; (2) Koreans highlighted promotions and firms related to processed products; and (3) both countries showed family orientation/domesticity and sentiment/emotion [19,20].

Comments from Mexico’s Starbucks Facebook page (a shop chain that sells organic coffee) reflected that people interacted more through happiness, but anger and longing were often used to generate participation [21]. Predicted effects of comments and likes were found when comments in Facebook posts related to organic food were perceived as useful, and the number of likes had an effect on negative emotions and willingness to pay [22]. A study involving organic local food suggested that such products can gain popularity and overcome purchasing barriers (eg, high price, low awareness, and low availability) through integration in consumers’ daily experiences with Facebook [23].

However, video networks related to organic or local food have not been thoroughly researched. Video social networks account for over half of the internet traffic when measured in bytes [24], and YouTube is an integrated video sharing platform that tends to form small-world networks [25]. An analysis of 76 food safety–related videos posted on YouTube found that artful quality was important to attract viewers and that the intended purpose of the videos can differ from the viewers’ perception of them [26]. Moreover, a study in Europe argued that YouTube was the least used media for consultation in case of food risks, although its general usage was high, particularly among younger generations [27]. In summary, communities related to sociotechnical food systems can be observed through video social networks in order to understand their communication patterns and discourses.

## Methods

### Operational Definitions

There is no consensus on what is organic or local food. A definition adapted from previous reports [28,29], establishes that any foods produced, processed, and managed through methods that do not implicate harmful synthetic inputs or additives, irradiation, or genetically modified organisms in compliance with standards generally set by the country in which they are sold, are considered organic.

With regard to local food, the most recognized feature is the marketing arrangement [30]. Thus, local food can be defined as food sold by producers to consumers and food sold by producers directly to retailers at markets or other regional areas..

### Data Collection

According to applicable institutional and national guidelines and regulations, ethics approval was not required for this study, as we focused on publicly available YouTube data. Video data were extracted in 2015 with YouTube Data Tools [31], which use the YouTube Data application program interface (API). The following two modules were employed: Video Network and Video Info. The first module builds a network starting with a query (one or more keywords), thus retrieving related videos and their metrics (number of views, likes, dislikes, comments, and categories). It also creates a graph file readable through the Gephi software [32]. As for the second module, video identifiers obtained through the first module were used to extract video comments.

### Classification of Data

The following three queries were used to extract video data: “organic food,” “local food,” and “local organic food.” The resulting three files were appended into one. Videos that appeared only with the “organic food” query were labelled as “organic food videos.” There were no videos that appeared exclusively with the “local food” query; thus, all videos that were not labelled as “organic food videos” were labelled as “local organic food videos.” Videos were watched by an investigator, and in case the content was not in an understandable language, the investigator requested a native speaker to interpret the content. Content that clearly was unrelated to food was discarded, reducing a total of 964 videos to 923 videos. The videos were also classified according to country and uploader. The types of uploaders considered were as follows:

- Business: It included businesses related to food production, processing, and distribution. Businesses linked to health, tourism, and banking were added to this category as well.
- Community: It included citizens and communities.
- Education: It included citizens disclosed as professors, researchers, students, and lecturers; research institutions; universities; and informal education-related accounts.
- Media: It included both traditional and internet-based media.
- Others: It included government, politicians, and celebrities.
- Undisclosed: It included all accounts that did not fit in the previous categories.

### Framing Analysis

Video content was also classified according to food frames. Framing is the action of using images and words to influence how audiences interpret a message, promoting specific versions of reality. They are useful tools to infer what people think is important.

Food-related framing usually falls into the corporative-political category [33] or into the social activism category [34]. Nonetheless, this study employed van Gorp and van der Goot [35] framing categories, which are used by stakeholders of sustainable food products and are based on archetypes (Table 1). The “Natural Goodness” frame and the “Good Mother” frame are used frequently and are strongly interlinked [35]. Therefore, most of the videos were classified in two or more frame categories, taking into account discursive/textual and visual cues. Only videos that reflected at least one frame were employed in further analysis.

**Table 1:** Condensed frame packages for sustainable food products.

Frame	Emotional basis	Key concepts	Visual cues	Textual cues
Responsibility	Endearment	Accountability and vulnerability	Children, fragile plants, and young animals	Caring, future generations, our children, and vulnerability
Undermining of foundations	Alarm and concern	Balance, base, complex systems, and links	Interconnections between elements in the ecosystem	Fragile balance, mutual dependency, and unstable
Frankenstein	Anxiety and unscrupulousness	Apocalypse, Pandora’s box, and sorcerer’s apprentice	Monsters and skulls	Frankenstein food, poison, and risks
Natural goodness	Admiration and astonishment	Authenticity, good taste, health, and purity	Idyllic nature and products	Natural, pure, and taste
Progress	Trust	Modernization and progress	High-tech tools	A better world, constant striving, and technology
Good mother	Gratitude, enjoyment, and love	Freedom of choice and great variety of products	Pleasure of shopping and rich harvest	Friendliness and product range

### Social Network Analysis

This type of analysis involves theories, methods, and techniques to study social relations and their structures [36]. A social network can be defined as a group of nodes and ties, where a node represents an entity (a YouTube video in this case) and a tie represents a relationship. In this study, ties connect nodes to related videos. Social network measurements were made with the Gephi software package.

### Sampling and Cleaning of Comments

Based on the study by Tsou et al [37], a random sample of comments made on “average” videos was considered to conduct semantic and sentiment analyses. In other words, comments from videos that had a maximum of 500 and a minimum of 5 comments were sampled. Thus, a total of 213 videos (155 from the organic network and 58 from the local organic network) were selected for sampling. The number of total extracted comments for each video was identified and included in a random number generator [38]. The outcome was five numbers that were used to select the comments, resulting in a total of 1065 comments. If a comment was not in English, the random number generator was employed again until a comment in English was selected. Moreover, text belonging to 13 comments not retrieved completely by the YouTube API was added by hand. URLs (web addresses) were shortened, as they were not the focus of this study. Information on replies to Google+ and Twitter accounts was also omitted.

### Semantic Analysis

This is a type of network study where the unit of analysis is keywords. The software employed was TI, an open source tool that generates a word frequency list, a word-occurrence matrix, a word co-occurrence matrix, a normalized co-occurrence matrix, and a word list from a set of short texts [39]. In order to cope with semantic ambiguities, coding notes were employed and refined over multiple test rounds to adjust the data, especially in the case of frequently found synonyms and plurals. Because TI does not recognize emoticons or punctuation symbols, these features were later included in the sentiment analysis.

### Sentiment Analysis

The sentiment analysis aimed to determine the polarity of text through natural language processing. Although most sentiment studies on social networks do not consider emoticons, this tendency has reverted in recent years, as their inclusion increases accuracy. Thus, the value of emoticons (positive, neutral, or negative) was assigned based on the SentiStrength software package [40] and in the context of comments sampled for this study.

## Results

### Classification of Videos and YouTube Metrics

Among the 923 videos related to organic and local food, 448 were included in the organic food video list and 475 in the local organic food video list. Overall, 606 videos disclosed location (47 countries). As the keywords employed were in English, the lists contained videos mostly from the United States (n=393). The second most frequent location was undisclosed (n=317), followed by India (n=25) for organic food videos and Canada (n=39) for local organic food videos. Overall, organic food videos had higher metrics in terms of views, likes, dislikes, and comments (Table 2).

As for types of uploaders, media-related YouTube channels were the most common for both lists (Table 3). In the case of organic food videos, the second most frequent uploader was “Education,” whereas in the case of local organic food videos, it was “Community.” However, the differences were not significant (*P*=.42).

**Table 2:** Nonparametric test of YouTube metrics.

Metric and group		N	Mean rank	Sum of ranks	Z
**Views**		923			11.056^a^
	Organic	448	561.90	251729.00	
	Local organic	475	367.78	174697.00	
**Likes**		905			12.655^a^
	Organic	441	565.08	249199.50	
	Local organic	464	346.48	160765.50	
**Dislikes**		905			8.319^a^
	Organic	441	515.27	227234.00	
	Local organic	464	393.82	182731.00	
**Comments**		899			9.304^a^
	Organic	436	528.26	230323.00	
	Local organic	463	376.30	174227.00	

^a^*P*<.001.

**Table 3:** Types of uploaders of organic and local organic food videos.

Uploader	Organic^a^ (N=448)	Local organic^a^ (N=475)
Business	37	87
Community	78	94
Education	93	74
Media	194	157
Others	7	17
Undisclosed	39	46

^a^χ^2^_1_ (N=923)=4.15; *P*=.42.

The top organic-related videos in terms of views and likes were uploaded mostly by media channels and contained short educative facts about food products. However, some of them were sensationalist. The top video was “Grocery Store Wars,” a stop-motion parody of the movie franchise Star Wars, contrasting organic food products and conventional food products in a supermarket. On the other hand, the top videos related to local organic food were business oriented and sometimes employed humor. The video with the highest number of views and likes was a three-dimensional animation commercial, which was part of a campaign by Chipotle Mexican Grill, an American restaurant chain. This video contrasted chemically treated and mechanically processed food products with food produced and processed by farmers. It can be concluded that the top videos in both cases were story-telling driven and showed a contrast between sustainable and nonsustainable foods.

### Frames in Organic and Local Organic Food Videos

Based on van Gorp and van der Goot [35], the Good Mother and Natural Goodness frames were expected to be found most frequently in video images and narratives. The third most used frame was Undermining of Foundations in both video groups. A difference was found for the fourth most used frame, which was Frankenstein in the case of organic food videos and Responsibility in the case of local organic food videos (Table 4).

**Table 4:** Types of frames in organic and local organic food videos.

Frame	Organic^a^ (N=808), n (48.26%)	Local organic^a^ (N=866), n (51.73%)
Good mother	282 (34.9%)	311 (35.9%)
Natural goodness	220 (27.2%)	253 (29.2%)
Undermining of foundations	153 (18.9%)	180 (20.7%)
Frankenstein	68 (8.4%)	27 (3.1%)
Responsibility	42 (5.1%)	57 (6.5%)
Progress	43 (5.3%)	38 (4.3%)

^a^χ^2^_1_ (N=1674/923)=4.84; *P*=.03.

### Comparison of Network Metrics and Structures

**Figure 1:**
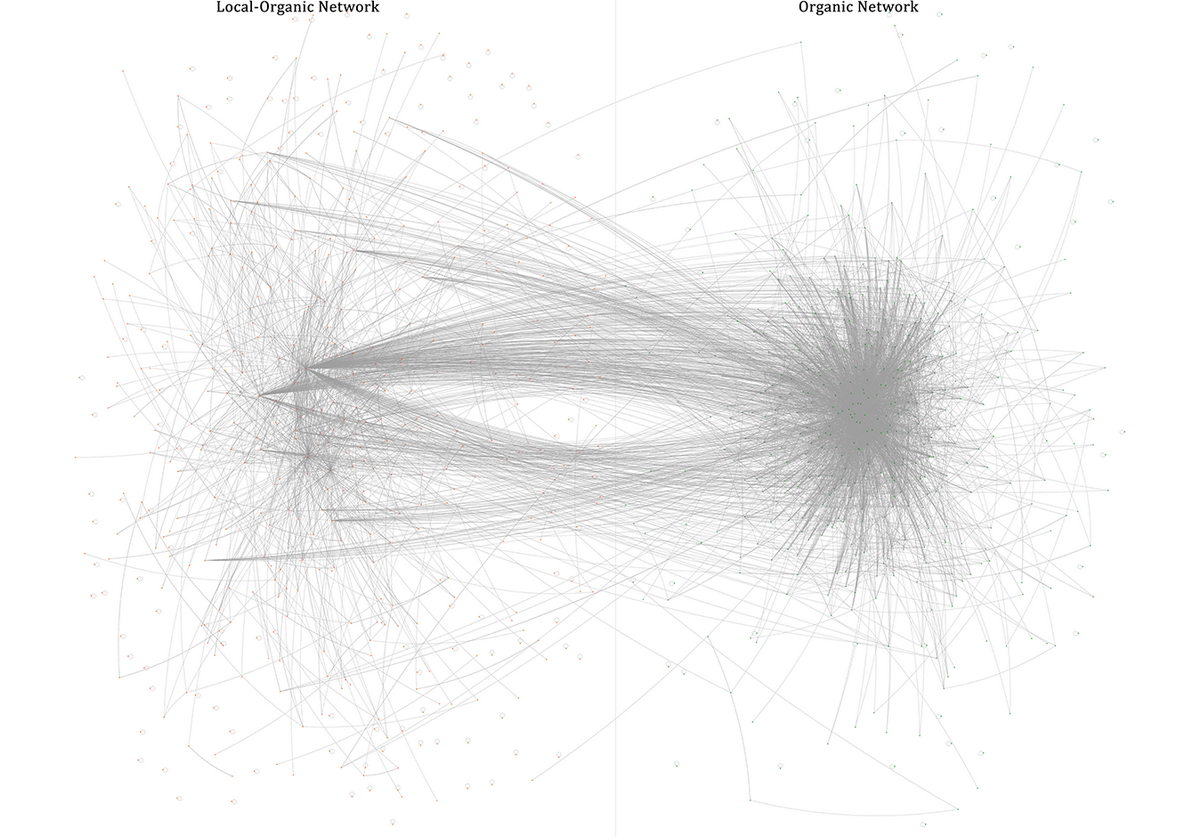
The local organic food video network and the organic food video network tied together. The organic food video network is presented as a star with central videos reaching the most distant videos within the network.

In order to visualize how different is the structure of the two video groups, their network metrics were compared. Because both lists share ties, metrics for the entire video network were also calculated. Local organic food videos had higher connected components, modularity, diameter, and average path lengths (Table 5). Density, number of shortest paths, degree, clustering coefficient, betweenness, and closeness were lower. Such results indicated that the local organic food network was less consolidated than the organic food network. On adding videos from the organic food list, the network showed a slight decrease in its diameter and became better connected, suggesting a broadcast typology for the organic food video network. This can be seen in Figure 1, which is drawn with NodeXL [41].

**Table 5:** Network centralities.

Centrality name	Description	Organic network	Local organic network	Organic and local organic network
Weakly connected components	Subgroups of nodes that can be reached from every other node in the group.	48	155	197
Density	Total number of ties divided by the number of all possible ties that can exist within a network.	0.038	0.005	0.007
Modularity	The strength of the division between subgroups in a network.	0.197	0.471	0.329
Diameter	Average of the maximum distance between the nodes of a network.	12	16	15
Path length	Average of the distance between the nodes of a network	3.902	4.640	4.652
Number of nodes	N/A^a^	448	475	923
Number of shortest paths	N/A	95,908	28,967	263,249
Degree	Average number of direct connections a node has to other nodes.	9.67	2.50	6.75
Clustering Coefficient	Measure of how close a node is to be part of a group.	0.191	0.081	0.142
Closeness	Average number of steps to access all the other nodes in a network.	3.272	2.509	2.287
Betweenness	Number of shortest paths that connect other nodes in the network by passing through a specific node.	621.457	222.01	532.013

^a^N/A: not applicable.

The top videos in terms of betweenness and in-degree centrality explained the basics of organic and local food and were predominantly uploaded by media channels. In contrast, videos with high out-degree centrality were uploaded mostly by businesses and individuals. A few organic-related videos identified the food as expensive, while local organic-related videos usually presented a specific area where such food products were available.

Spearman correlation analyses between YouTube metrics and network-related metrics were performed to find how much the popularity features are related to communication patterns in the network. The number of views, likes, dislikes, and comments were moderately correlated with degree, modularity class, eigenvector, and betweenness centralities in the organic food video network (Table 6). As for the local organic network, there were less correlations and the ranks were lower (Table 7). This supports the assumption that the organic network has more consolidated features.

**Table 6:** Spearman correlations for the organic network.

Variable	Degree	Modularity class	Clustering coefficient	Betweenness
Views	0.209^a^	0.174^a^	−0.185^a^	0.218^a^
Likes	0.187^a^	0.192^a^	−0.201^a^	0.182^a^
Dislikes	0.240^a^	0.120 (*P*=.01)	−0.180^a^	0.193^a^
Comments	0.182^a^	0.157 (*P*=.001)	0.191^a^	0.164 (*P*=.001)

^a^*P*<.001.

**Table 7:** Spearman correlations for the local organic network.

Variable	Degree	Modularity class	Clustering coefficient	Betweenness
Views	0.156 (*P*=.001)	0.116 (*P*=.01)	0.015 (*P*=.75)	0.287^a^
Likes	0.087 (*P*=.06)	0.075 (*P*=.11)	−0.030 (*P*=.52)	0.239^a^
Dislikes	0.086 (*P*=.07)	0.039 (*P*=.41)	0.029 (*P*=.54)	0.177^a^
Comments	0.143 (*P*=.002)	0.081 (*P*=.08)	0.045 (*P*=.34)	0.257^a^

^a^*P*<.001.

### Semantic Analysis of Comments

**Figure 2:**
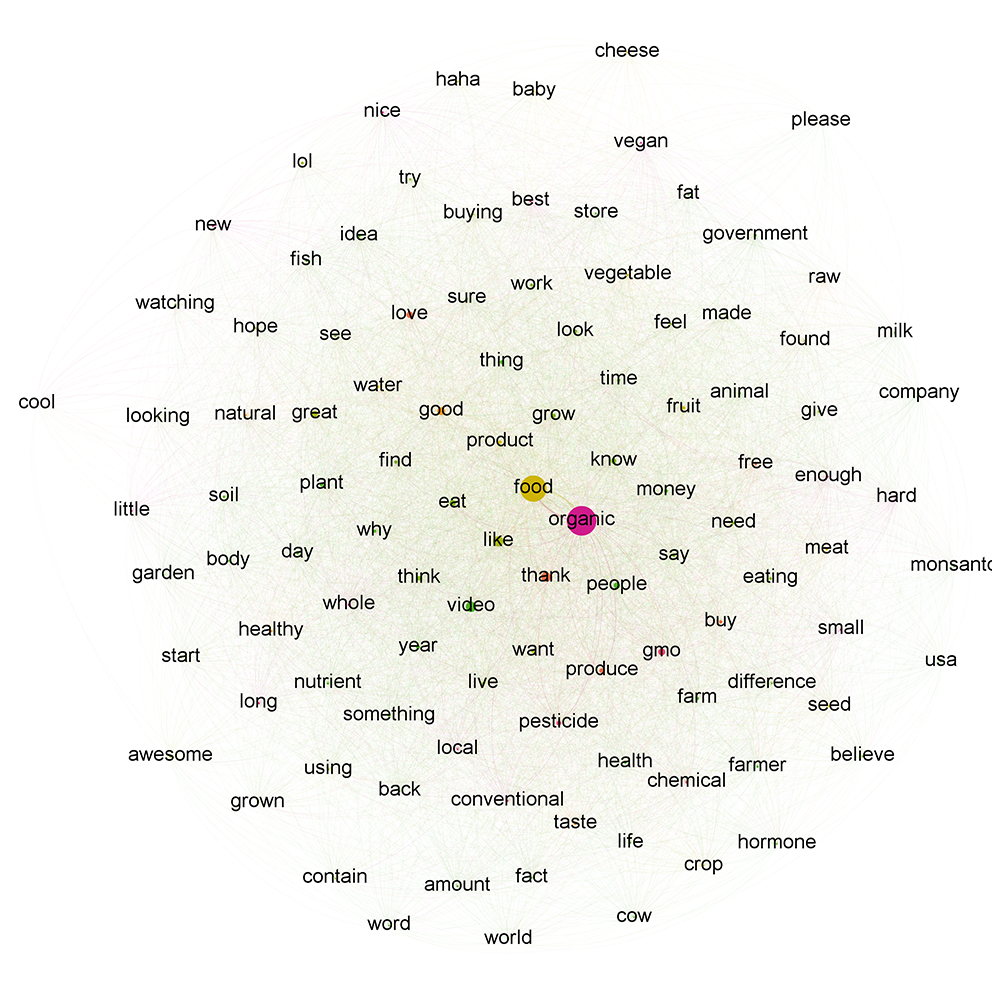
Semantic network for organic food video comments. The size reflects the word frequency. Ties show which words were found in the same comment, with tie thickness reflecting the frequency of such relationships.

The Gephi software was used to visualize the semantic networks corresponding to the two video lists. The 107 most frequent words found in the comments sample from the organic food videos were represented with nodes (Figure 2).

**Figure 3:**
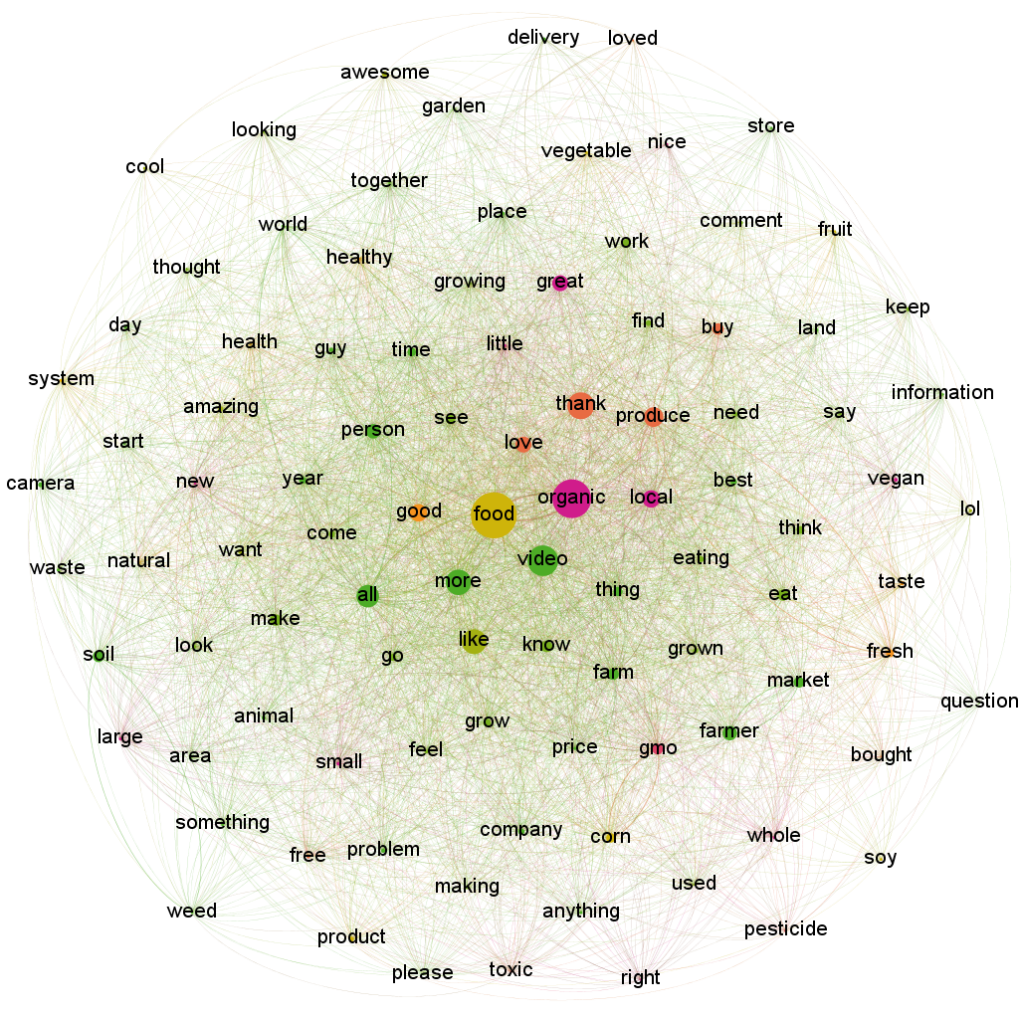
Semantic network for local organic food video comments.

The word “organic” was the most frequent in these comments, with the term “food” closely related to it. Verbs connected to “organic” were “grow,” “know,” “like,” “need,” “say,” and “think.” Frame-related words are presented in Table 1. Textual cues associated with the Good Mother frame were “love,” “product,” and “thanks.” Words connected to the Natural Goodness frame were “health,” “healthy,” “raw,” and “taste.” Textual cues associated with the Frankenstein frame were “chemical,” “gmo” (genetically modified organism [GMO]), “hormone,” “Monsanto,” and “pesticide.” All terms related to this frame were connected to “organic.” Products included fruits, vegetables, fish, meat, cheese, and milk. Positive words included “awesome,” “cool,” “great,” “hope,” “nice,” and laughing textual representations like “haha” and “LOL.” The 98 most frequent terms found in the comments sample extracted from the local organic food videos were mapped (Figure 3). The word “food” was the most frequent in these comments, with the words “good,” “organic,” and “video” closely related to it. The verbs connected to “local” were “eating,” “love,” “need,” “produce,” “think,” and “know.” Textual cues associated with the Good Mother frame were “love,” “product,” and “thanks.” Words related to the Natural Goodness frame included “fresh,” “health,” “healthy,” “natural,” and “taste.” In particular, the terms health and healthy were found less frequently than in the organic-related comments. Textual cues associated with the Frankenstein frame were “gmo,” “pesticide,” and “toxic.” Although both the words “local” and “organic” were connected to “gmo,” only the word “organic” was connected to “pesticide” and “toxic,” suggesting a more frequent usage of this frame regarding organic food. Products included fruits, vegetables, corn, and soy. The positive terms were “awesome,” “cool,” “nice,” and laughing textual representations like “LOL.”

In summary, while both groups of comments featured fruits and vegetables, organic food videos featured dairy and proteins, and local organic food videos featured grains and cereals. Moreover, the organic food network had a greater variety of Frankenstein frame–related words.

### Sentiment Analysis of Comments

There was no relevance with regard to negativity valence. However, comments made on local organic videos had a higher rank for positive valence in comparison with comments made on organic videos (Table 8).

**Table 8:** Nonparametric test: sentiment valence of comments made on the videos.

Valence and group		Mean rank	Sum of ranks	Z	*P*
**Positive valence**				2.159	.03^a^
	Organic	521.2	403927		
	Local organic	564.54	163718		
**Negative valence**				0.15	.88^a^
	Organic	532.27	4125409		
	Local organic	534.95	155136		

^a^N_1_=775, N_2_=290.

## Discussion

### Principal Findings and Comparison With Prior Work

Organic and local food products were communicated on YouTube by several actors articulating efforts to educate the public about food. In particular, the position of organic food videos in the network strengthened the diffusion paths, with a better structural capital than local organic videos, whereas the high in-degree (ties directed to a video) suggested a broadcast network. The relationship between network metrics and negative reactions (number of dislikes and less positive sentiment in comments) implied that negativity might play a relevant role in the diffusion of videos. This coincides with the theory that negative relationships might explain social network outcomes better than positive relationships [42], although most of the video content and user reactions fell in the positive category. Other factors like psychological traits, which influence food choice [43], should also be taken into account. The interplay between psychological traits and the communication of negative/sensationalist video content related to sustainable food should be investigated further in future studies.

Another relevant finding was the words related to “organic” and “local.” The frames have been summarized in Table 1. Although both terms were used in contrast with “gmo,” the amount of risk and artificial-related words linked to “organic” was larger. Taking into account that “organic” and “local” are viewed as good food alternatives (in congruency with the frequent usage of the Natural Goodness and Good Mother frames), a clear contrast between what is considered dangerous and artificial can be seen in some video-related comments. Further, there was more emphasis on health among these comments than in the local organic–related text, reflecting a relationship with subjective well-being, and it is moderated by health concern that has been documented in scientific literature previously [44].

Words like “love” and “thanks” were used often in the case of comments for local organic videos. As they also employed the Responsibility frame more frequently, it implies a more human and social dimension for the word “local.” It also has implications for activating the viewer in areas besides food consumption, as images involving future generations and nonhuman living beings are part of this frame. This partly explains why comments for local organic videos were more positive than those for organic videos.

Finucane et al [45] argued that individuals who have favorable opinions about GMOs are more likely to endorse statements reflecting hierarchical views. The communities analyzed in this study might then represent an egalitarianism perspective linked to organic and local food. Commenters shared their own research on the topics in an articulated, generous, and respectful voice. Thus, usage of the words “know,” “think,” “please,” and “thanks” was frequent.

The persistence of uncomfortable feelings among the public towards GMOs, which are considered as “monsters” according to the Frankenstein frame, points to a failure in scientific communication. This is particularly true regarding information on pesticides, soil depletion, and nutrition. Such communication patterns could be improved by closing the gap between scientists and the public, as attempted by some of the videos uploaded by education channels. This could potentially bring more transparency and trust to the food production chain.

Dissimilar ideas of what is food influence the outcomes of guidelines and food policies. There is little knowledge on the effect of YouTube’s ranking criteria in areas other than politics and entertainment. Hence, a multimethod analysis of the communication of basic human needs, such as food, can provide us with more understanding of the consequences of algorithm usage in rich social media and its interplay with human users. Moreover, we uncovered communication patterns and specific visual and textual cues, providing more comprehensive insights of a complex ecosystem of actors involved in food production, distribution, and consumption. The case of organic and local food intertwines consumerism with environmental concerns that have the potential to impact public health.

### Limitations

As the language of analysis was English, there was partially limited access to videos from non-English speaking cultures. Sentiment can be influenced by many contextual features, such as weather [46] and geographical location [47], which were not taken into account for this study.

### Conclusions

This study explored the communication paths, discourses, opinions, attitudes, behaviors, and emotions related to sustainable food products in YouTube video networks. Based on our objectives, we can make the following conclusions:

- The organic video network was more consolidated than the local organic video network and was driven mostly by media and business YouTube channels.
- The “Good Mother” and “Natural Goodness” frames were most frequently employed in the videos, followed by “Undermining of Foundations.”
- The concept “organic” has become consolidated among both specialists and the public, while the term “local” is in the process of acquiring a formal definition. Nevertheless, the term “organic” was slightly more associated with health risks and negative feelings, while the term “local” was perceived as more human/social and more positive.

Further studies could incorporate more languages, as well as a larger data set. Time-based analysis and segmentation of semantic analysis across different geographical locations would also deepen the present understanding of the diffusion of both organic and local food products. Another area for further exploration is GMOs, as this technology will continue to transform food production and consumption patterns in contrast with traditional agricultural methods.

## Abbreviations

API: application program interface
GMO: genetically modified organism
